# Analytical validation of a direct lipoprotein(a)-cholesterol assay

**DOI:** 10.1016/j.jlr.2026.101008

**Published:** 2026-02-23

**Authors:** Santica M. Marcovina, Spenser Smith, Lizhu Lin, Sotirios Tsimikas

**Affiliations:** 1 Medpace Reference Laboratories, Cincinnati, OH, USA; 2 Vascular Medicine Program, Division of Cardiovascular medicine, University of California San Diego, La Jolla, CA, USA

**Keywords:** cholesterol, LDL, lipoprotein(a), cardiovascular, diagnosis, therapy

## Abstract

Accurate measurement of lipoprotein(a)-cholesterol [Lp(a)-C] may be useful in interpreting the traditional lipid panel, particularly in patients with high Lp(a). We developed and analytically validated a direct immunocapture ELISA in a Clinical Laboratory Improvement Amendments-certified laboratory for quantifying Lp(a)-C in human plasma using an apolipoprotein(a)-specific monoclonal antibody (LPA4) coupled to magnetic beads. The linearity of the assay was found to be excellent (*R*^2^ = 1.00), with % bias ranging from 0.3% (upper limit of quantification) to 16.1% (lower limit of quantification) and coefficient of variation ranging from 3.4% to 16.2%. The analytical measuring range was 0.78 mg/dl to 40.0 mg/dl, and the limit of blank and limit of detection were 0.02 mg/dl and 0.05 mg/dl, respectively. The intra- and interassay coefficients of variation, determined on four quality control samples, ranged from 4.9% to 7.6% and from 12.6% to 15.0%, respectively. No interference was observed from hemolysis (up to 0.71 g/dl), bilirubin (up to 10.1 mg/dl), or triglycerides (up to 1,082 mg/dl. Lp(a)-C was stable for at least 3 months at −70 °C and through two freeze-thaw cycles. Direct Lp(a)-C correlated strongly with Lp(a) molar concentration (*r* = 0.93, *P* < 0.001). This study reports the results of the first analytical validation of a direct method to quantify Lp(a)-C, enabling standardized quantification of Lp(a)-C suitable for research studies and clinical trials; additional clinical outcome validation will be required prior to routine clinical implementation.

Lipoprotein(a) [Lp(a)] is a genetically determined, independent risk factor for atherosclerotic CVD (1). Lp(a) is an LDL-like particle that contains cholesterol within its lipid core, as demonstrated by classical ultracentrifugation and biochemical compositional studies (2, 3). Prior measurements have shown that the cholesterol content associated with Lp(a) [Lp(a)-C] varies across individuals (4).

All currently reported LDL-C results, whether obtained by ultracentrifugation, direct homogeneous assays, or estimation formulas, include the cholesterol contained within Lp(a) particles (5). In individuals with high Lp(a), Lp(a)-C can contribute clinically relevant amounts of cholesterol to measured LDL-C (4, 6). This issue is particularly relevant in research and clinical trial settings where both LDL-C and Lp(a) may be simultaneously modified by treatment, underscoring the need for precise partitioning of cholesterol components. Because Lp(a)-associated cardiovascular risk exists on a continuum in its association with atherosclerotic CVD risk (7), accurate quantification of Lp(a)-C may be useful in interpreting the effects of both LDL-C- and Lp(a)-lowering therapies. HDL-C represents a separate cholesterol-containing lipoprotein fraction within the plasma lipid profile, whereas HDL-C does not contribute to Lp(a)-C measurement. The partitioning of cholesterol among lipoprotein classes is relevant when interpreting the contribution of Lp(a)-C to total cholesterol and LDL-associated cholesterol.

Despite recognition of this analytical gap, no direct, validated, high-throughput method for Lp(a)-C quantification has been widely implemented. To address this unmet need, we previously developed and optimized a sensitive, high-throughput, immunocapture-based assay for direct quantification of Lp(a)-C (4). The assay demonstrated high specificity, with no detectable signal in plasma from individuals genetically lacking Lp(a). Here, we report the analytical validation of this method to establish its precision, linearity, and robustness and to provide preliminary estimates of the distribution of Lp(a)-C values in a cohort of healthy donors, supporting its implementation in future studies.

## Materials and methods

### Antibodies

The direct Lp(a)-C immunocapture ELISA uses the recombinant murine monoclonal IgG antibody LPA4 (8). This antibody recognizes the 14-amino acid peptide TRNYCRNPDAEIRP present on apo(a) KIV_5_, KIV_7_, and KIV_8_ and the partial sequence NYCRNPDA present on KIV_2_, ensuring high affinity and specific capture of Lp(a) without crossreactivity with apoB or plasminogen. Although one of the recognized epitopes is located within KIV_2_, this monoclonal antibody is used in the assay to capture whole Lp(a) particles regardless of apo(a) isoform size. Because cholesterol is quantified after complete particle capture and immunoprecipitation, the measured Lp(a)-C reflects total cholesterol mass within the captured particle population, and therefore, the accuracy of the assay is not impacted by the variable number of apo(a) KIV_2_ repeats.

### Determination of Lp(a) molar concentrations and laboratory variables

Lp(a) particle numbers in nmol/l were measured using the Roche Tina-quant Gen.2 kit on a Roche c502 analyzer. Each of the five standards used to calibrate the assay is formed by plasma pools with apo(a) isoforms ranging from large to small and Lp(a) concentrations from low to high. The target values for each standard were independently assigned against the World Health Organization/International Federation of Clinical Chemistry and Laboratory Medicine SRM-2B reference material, thus resulting in minimized impact of apo(a) isoform size-generated bias. Direct LDL-C concentration was determined by a homogeneous enzymatic method using the Roche reagent and a calibrator on a Beckman Coulter analyzer. Total cholesterol was determined using the Beckman reagent on a Beckman Coulter analyzer.

### Methodology for Lp(a)-C quantification

**Figure 1:**
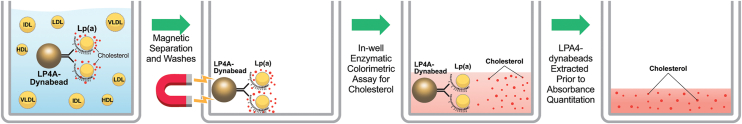
Methodology of the Direct Lp(a)-C assay. Immunocapture of Lp(a) particles is accomplished with a monoclonal antibody recognizing apolipoprotein(a) coupled to magnetic beads, a washing step to remove nonspecifically associated lipoproteins and measurement of total cholesterol with a colorimetric reagent.

The direct Lp(a)-C assay is based on the predicate method developed in the Tsimikas/Yeang Laboratory at the University of California San Diego (UCSD) (4) (Fig. 1). Lp(a)-C concentration refers to the mass concentration of cholesterol contained within immunocaptured Lp(a) particles in plasma (mg/dl), rather than percent particle composition. In brief, plasma samples are first incubated with magnetic beads covalently coupled to LPA4 to isolate Lp(a) particles from other plasma lipoproteins. Because nonspecific pulldown of other lipoproteins may occur and artifactually increase the measured cholesterol content, an approach was developed to remove nonspecific lipoprotein binding by adding proline (Sigma-Aldrich, St Louis, MO) and epsilon aminocaproic acid (Acros Organics, Geel, Belgium) to the buffer solution. After multiple washing cycles to eliminate nonspecifically bound lipoproteins, the captured Lp(a) particles remain immobilized on the magnetic beads. These particles are then subjected to cholesterol quantification using a standard enzymatic colorimetric assay, which measures total cholesterol content by enzymatic conversion of cholesterol to a detectable colored product read spectrophotometrically using a BioTek Synergy HTX plate reader. The resulting Lp(a)-C concentration is reported in mg/dl (or mmol/l) and reflects only the cholesterol content within the immunocaptured Lp(a) particles. Conditions were established to optimize the amount of beads and plasma dilution to ensure complete capture and immunoprecipitation of Lp(a) so that the total Lp(a)-C content, after correction for the plasma dilution, is reflective of the total concentration in plasma (4).

The following method for measuring the total cholesterol (free + esterified) content on the immunocaptured Lp(a) is briefly summarized as follows: cholesteryl esters, which typically constitute 60–70% of circulating cholesterol, are hydrolyzed to free cholesterol and fatty acids by cholesterol esterase. Next, free cholesterol is oxidized by cholesterol oxidase, producing cholest-4-en-3-one and hydrogen peroxide as a byproduct. The generated hydrogen peroxide reacts with 4-aminoantipyrine and a phenolic compound via horseradish peroxidase to form a quinonimine dye, which develops a color measurable by spectrophotometry at 500 nm. The intensity of the color formed is directly proportional to the total cholesterol concentration in the sample. Calibration with cholesterol standards allows quantification of Lp(a)-C in mg/dl. In prior analyses (4), specificity was confirmed by the absence of measurable Lp(a)-C in plasma from individuals with extremely low Lp(a) (median, 0.8 mg/dl).

### Analytical validation of the direct Lp(a)-C assay

The UCSD direct Lp(a)-C method was transferred to Medpace Reference Laboratories, and the analytical validation was performed in accordance with the Clinical Laboratory Improvement Amendments (CLIA) and College of American Pathologists (CAP) accreditation requirements. Testing procedures were based on the Clinical and Laboratory Standards Institute method evaluation standards. Acceptability criteria for quantitation limits as well as storage and freeze-thaw stability were based on the 2022 Food and Drug Administration (FDA) M10 Bioanalytical Method Validation and Study Sample guidance.

### Sample handling

Venous blood samples were collected from fasting donors using standardized procedures. For plasma, blood was drawn in K_2_-EDTA tubes and centrifuged at 1,500 *g* for 10 min at 4°C to separate plasma. For serum, blood was collected in clot-activator tubes, allowed to clot at room temperature for 30 min, and centrifuged under the same conditions as above to separate serum. All samples were aliquoted and stored at −80°C until analysis. Prior to assay use, samples were thawed and thoroughly mixed by inversion to ensure homogeneity. All specimens were centrifuged at 16,000 *g* for 3 min to remove any residual particulates or cryoprecipitates. Only the clarified supernatant was used in the immunocapture assay.

### Precision: repeatability (within-run precision) and reproducibility (total imprecision)

Repeatability of the Lp(a)-C assay was assessed by measuring multiple replicates (n = 12) of four quality control samples containing low, medium, and high Lp(a)-C concentrations within a single analytical run using the same instrument, operator, and reagent lot. For assessment of reproducibility, the four quality control samples evaluated during repeatability testing were analyzed in duplicate at the beginning and end of each plate for 6 days. The assay’s repeatability and reproducibility were considered acceptable if the calculated percent of coefficient of variation (%CV) was ≤15.0%, consistent with Clinical and Laboratory Standards Institute method evaluation recommendations, CLIA/CAP-accredited laboratory performance standards, and FDA M10 bioanalytical method validation guidance for ligand-binding assays.

### Linearity and analytical measuring range

The analytical measuring range (AMR) was assessed by determining the accuracy and precision throughout a range of Lp(a)-C levels and determining the limit of detection (LOD), limit of blank (LOB), lower limit of quantitation (LLOQ), and upper limit of quantitation (ULOQ). For linearity, seven calibrators spanning the AMR were prepared and run in duplicate six times on 6 different days. Based on the FDA’s M10 Bureau of Medical Verification and Scientific Services of America (BMVSSA), the results were acceptable if the measured concentration at each level was within ±15.0% of the expected mean and the %CV was ≤15.0%.

Testing for LLOQ and ULOQ was incorporated into the linearity step by evaluating the performance of the levels at the limits of the AMR. Per the FDA 2022 M10 BMVSSA, the LLOQ, or “functional sensitivity,” is defined as the lowest concentration of an assay that can be measured with an accuracy of ±25.0% and total reproducibility of ≤25.0%. The ULOQ is defined as the highest concentration of an assay that can be measured with an accuracy of 25.0% and a total reproducibility of ≤25.0%. To determine the LOB and LOD, the sample dilution buffer was analyzed in 12 replicates in a single run. LOD was estimated as the mean ± 2 SD of the results from the 12 replicates of the blank.

### Assay accuracy—spike and recovery

Spike and recovery experiments were performed to identify systemic errors that may arise from interactions between other matrix components and the analyte of interest. Dilutability experiments were performed to confirm compatibility between the sample diluent and the matrix. The concentrations of Lp(a)-C in five low Lp(a) plasma samples were determined before (baseline) and after spiking with in-house Hi Lp(a)-C pool 3. The ratios of spike volume to total sample volume were 1:10 (1 part Hi Lp(a)-C pool 3 to 9 parts plasma) and 1:20 (Hi Lp(a)-C pool 3 to 19 parts serum). Samples were spiked before the recommended x2 dilution for low Lp(a) plasma samples. Duplicate measurements of each sample were performed, and the mean, SD, %CV, bias, and %bias were calculated. Recovery was deemed acceptable if the mean %bias was within ±20.0% of the baseline concentration (i.e., 80.0–120% recovery), consistent with FDA M10 bioanalytical validation guidance and Westgard Total Allowable Error benchmarks commonly applied in CLIA/CAP-accredited laboratory validation.

### Dilutability

The dilutability for Lp(a)-C is dependent on the Lp(a) molar concentration and categorized as low (Lp(a) <150 nmol/l), high (Lp(a) >150 to <500 nmol/l), and very high (Lp(a) >500 nmol/l). The concentrations of three plasma samples with Lp(a) <150 nmol/l (low) were determined before (baseline) and after diluting x2, x3, x4, x6, and x8 with the sample dilution buffer. The baseline concentration of two plasma samples with Lp(a) >150 but <500 nmol/l (high) was determined before and after diluting x8, x10, x12, and x20 with the sample dilution buffer. Finally, the baseline concentration of two additional plasma samples with Lp(a) >500 nmol/l (very high) was determined before and after diluting x15, x20, and x30 with the sample dilution buffer. Each sample was run in duplicate, and the mean, SD, %CV, bias, and %bias were calculated. Results were deemed acceptable if the measured concentration of the diluted sample was within ±20.0% of the baseline concentration after correcting for the dilution.

### Analytical specificity

Analytical specificity was assessed through the determination of interfering substances. Interferences from hemolysis (hemoglobin), lipemia (triglycerides), and icterus (bilirubin) were evaluated by spiking three normal human samples with varying levels of lysed whole blood (up to 1.14 g/dl of hemoglobin), triglyceride (intralipid up to 20,000 mg/dl), and bilirubin (up to 800 mg/dl) solutions. Samples were spiked with vehicle (saline for hemolysate and triglycerides and 0.1 N NaOH for bilirubin) at a volume equal to the volume used to spike the interferent and analyzed to establish the nominal concentration value of Lp(a)-C in the sample. Hemolysis, lipemia, and icterus were not considered to interfere until the bias was no longer within ±20.0% compared with the result of the sample spiked with vehicle.

### Stability assessment

Storage and freeze-thaw stabilities were evaluated using six fresh EDTA plasma samples from healthy volunteers. EDTA plasma was separated from blood within 1 h from blood collection and analyzed fresh on day 0. Aliquots were prepared and kept at various storage temperatures (ambient, refrigerated, frozen at −20°C, and frozen at −70°C). Samples stored at room temperature were analyzed every day for a total of 5 consecutive days. Samples stored at 2–8°C were analyzed on days 1, 2, 3, 4, 5, and 8. Samples stored at −20°C and −70°C were analyzed on day 1, week 1, week 3, month 1, and month 3. Evaluation of subsequent long-term storage stability at −20°C and −70°C is ongoing. Based on the criteria for manual ligand-binding assays, such as ELISA, specimens are considered stable until the mean %bias from the fresh sample is no longer within ±20.0%.

For freeze-thaw stability, six plasma samples were collected and processed as described above. Aliquots were prepared and taken through three freeze-thaw cycles at −20°C and −70°C. To ensure samples were adequately frozen, they were stored in the appropriate freezer for at least 12 h prior to thawing. At each thawing cycle, the aliquot was tested in duplicate.

### Plasma-serum correlation

Paired EDTA plasma and serum samples collected from 40 fasting, apparently healthy individuals were analyzed in parallel for Lp(a)-C. Results were evaluated using Deming regression and Bland-Altman analyses to determine bias. Results from serum and plasma were considered equivalent if no statistically significant bias was observed. The bias was not considered clinically significant if it was within ±20%.

### Distribution of Lp(a)-C in healthy normolipidemic donors

To characterize the distribution of Lp(a)-C in a healthy population, 94 EDTA plasma samples from fasting donors without significant clinical history were analyzed. Consistent with the known right-skewed distribution of Lp(a), both Lp(a) and Lp(a)-C values were non-normally distributed. Therefore, descriptive statistics, including median and interquartile range, are presented. No formal population reference interval was established, given the limited cohort size and the absence of demographic stratification.

### Statistical analyses

Continuous variables are reported as mean ± SD or median (interquartile range [IQR]) based on distributional characteristics. Validation data were analyzed using Analyse-it® (v5.68; Analyze-It Software Ltd), and acceptability criteria were based on FDA BM10 BMVSSA guidance and Westgard Total Allowable Error thresholds. The mean values of the assay comparison were analyzed using Deming regression and Bland-Altman (9) plots to determine bias. Normality was tested with the Shapiro-Wilk test. Spearman's correlations were used to analyze relationships between groups. Statistical analyses were performed using SPSS, v29.0 (IBM), with two-sided *P* < 0.05 considered statistically significant.

## Results

### Precision

#### Repeatability (within-run imprecision) and reproducibility (total imprecision)

For the repeatability study, the %CVs for the four quality control samples with Lp(a)-C concentrations of 5.8, 6.9, 32.0, and 39.0 mg/dl were 5.7%, 5.9%, 7.6%, and 4.9%, whereas for the reproducibility study, the %CVs were 12.6%, 14.4%, 13.0%, and 15.0%, respectively.

#### Linearity and AMR

**Figure 2:**
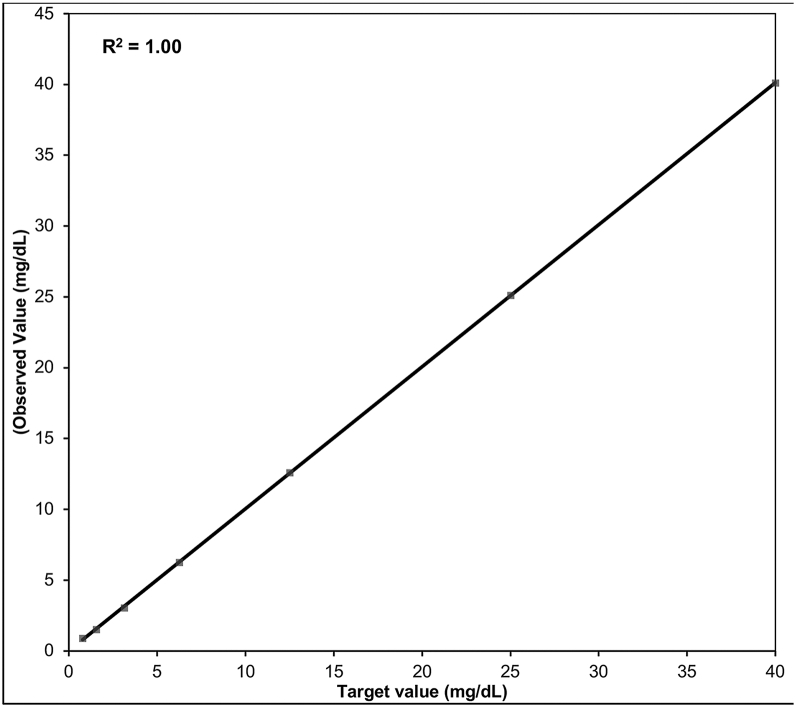
Linearity of the Direct Lp(a)-C assay. Results obtained by analyzing seven levels of calibrator solutions demonstrated the assay is linear from 0.78 to 40.0 mg/dL.

As evidenced in Fig. 2, the assay demonstrated excellent linearity (*R*^2^ = 1.00), and across the seven calibration levels, the bias ranged from −1.9% to 16.1% and %CV ranged from 3.2% to 16.2%.

### Lower and upper limits of quantitation for Lp(a)-C

The lowest standard solution that demonstrated acceptable performance at the LLOQ was 0.78 mg/dl (level 1), with an observed %bias and %CV (reproducibility) of 16.1% and 16.2%, respectively. The highest standard solution that demonstrated acceptable performance at the ULOQ was 40.00 mg/dl (level 7), with an observed %bias and %CV of 0.3% and 3.4%, respectively. The LOB was 0.0199 mg/dl, and the calculated LOD was 0.0455 mg/dl.

### Spike and recovery for Lp(a)-C

In the spike and recovery experiments, the %bias for the five samples ranged from −13.1% to 15.4%, which is well within the acceptable criteria of 20% (Table 1).

**Table 1:** Matrix effects and compatibility—spiking and recovery experiments

Run date	Baseline		Spike		Expected	Observed	Bias (%)
	Concentration (mg/dl)	Volume used (μl)	Concentration (mg/dl)	Volume used (μl)	Concentration (mg/dl)	Concentration (mg/dl)	%
Sample 1							
October 12, 2023	4.80	90	27.74	10	7.09	7.75	9.4
	4.80	190	27.74	10	5.95	6.53	9.8
Sample 2							
October 12, 2023	2.70	90	27.74	10	5.21	4.87	−6.5
	2.70	190	27.74	10	3.95	4.00	1.2
Sample 3							
October 12, 2023	2.85	90	27.74	10	5.34	4.87	−8.8
	2.85	190	27.74	10	4.09	4.72	15.4
Sample 4							
October 12, 2023	9.05	90	27.74	10	10.92	10.86	−0.6
	9.05	190	27.74	10	9.99	9.56	−4.3
Sample 5							
October 12, 2023	2.77	90	27.74	10	5.27	4.72	−10.4
	2.77	190	27.74	10	4.02	3.50	−13.1

### Dilutability for Lp(a)-C

#### Low Lp(a) results

All three samples showed acceptable recovery for x3, with the %bias from baseline (x2) for the three samples ranging from −1.3% to 5.7%. The x4 to x8 dilutions, ranging from 1.2% to 36.3%, were not within the 20% acceptance criteria.

#### High Lp(a) results

All three samples showed acceptable recovery for the x8 to x20 dilutions, with the %bias from baseline (x10) ranging from −12.0% to 12.4%.

#### Very high Lp(a) results

All three samples showed acceptable recovery for the x15 to x30 dilutions, with the %bias from baseline (x20) ranging from −7.0% to 7.0%.

### Analytical specificity

#### Hemolysis results

For all three samples tested, the %bias from the target concentration ranged from −16.7% to 27.1% at all concentrations up to 1.14 g/dl. No interference from hemolysis was detected at hemoglobin concentrations up to 0.71 g/dl.

#### Lipemia results

For all three samples tested, the %bias from the target concentration ranged from −19.7% to 27.5% at all concentrations up to 2,036.8 mg/dl. No interference from lipemia was detected at triglyceride concentrations up to 1,082.4 mg/dl.

#### Icterus results

The %bias from the target concentration for the recovery of Lp(a)-C ranged from −20.7% to 33.2% for all three samples at all bilirubin concentrations up to 40.56 mg/dl. No interference from icterus was detected at bilirubin concentrations up to 10.14 mg/dl.

### Stability assessment

Stability of Lp(a)-C in EDTA plasma was evaluated under various storage conditions (Supplement). At room temperature (18–25°C) and 4°C, samples remained stable for up to 4 h (%bias <20%), but significant degradation occurred by day 1 and day 7, indicating that longer storage under these conditions is unsuitable. It is therefore recommended that samples be processed and frozen at −70°C within 1 h of collection. When stored frozen, Lp(a)-C remained stable for at least 3 months at −20°C and −70°C, with a mean %bias relative to baseline within acceptable criteria. Freeze-thaw testing showed acceptable stability through two cycles at −70°C but exceeded acceptance criteria at −20°C after a single cycle.

### Plasma-serum correlation

**Figure 3:**
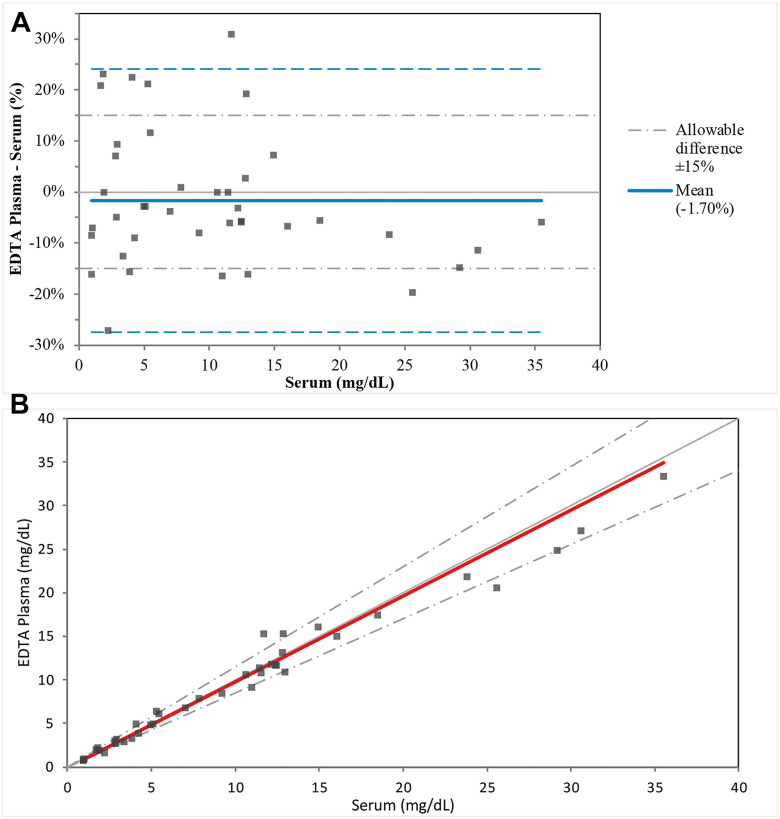
Assessment of bias observed between serum and EDTA plasma. A: Bland-Altman analysis and B: Deming Regression of EDTA plasma versus serum in determining Lp(a)-C.

Bland-Altman analysis identified a mean %bias of −1.7% between serum and EDTA plasma. Deming regression estimated the slope as 0.9854 and the intercept as −0.04265, with a good correlation coefficient (*R* = 0.988) (Fig. 3). No clinically or statistically significant bias was observed between serum and EDTA plasma.

### Range of Lp(a)-C, Lp(a), and lipid values in healthy donors

In the cohort of 94 subjects, Lp(a) concentrations covered a wide range with a mean (SD) of 93.7 (76.0) nmol/l and median (IQR) 80.4 (29.0–133.1) nmol/l (Table 2). The mean (SD) Lp(a)-C concentration was 5.4 (3.8) mg/dl (median [IQR], 4.4 [2.5–6.8] mg/dl). Among lipid measures, the mean (SD) direct LDL-C was 103.5 (25.0) mg/dl and total cholesterol was 177.5 (28.4) mg/dl.

**Table 2:** Lp(a) molar concentrations and measured and derived laboratory variables in 94 subjects

Variable	No. of subjects	Mean (SD)	Median (IQR)	Minimum	Maximum
Lp(a), nmol/l	94	93.7 (76.0)	82.6 (29.5–130.9)	9	339
Lp(a)-C, mg/dl	94	5.4 (3.8)	4.4 (2.5–6.8)	0.8	20.7
LDL-C, mg/dl	91^a^	103.5 (24.8)	104.1 (86.1–121.9)	39	157.1
Total cholesterol	91^a^	177.5 (28.4)	176.8 (157.0–196.3)	108	238.9

a Sample quantity was not sufficient for determination in three subjects.

**Figure 4:**
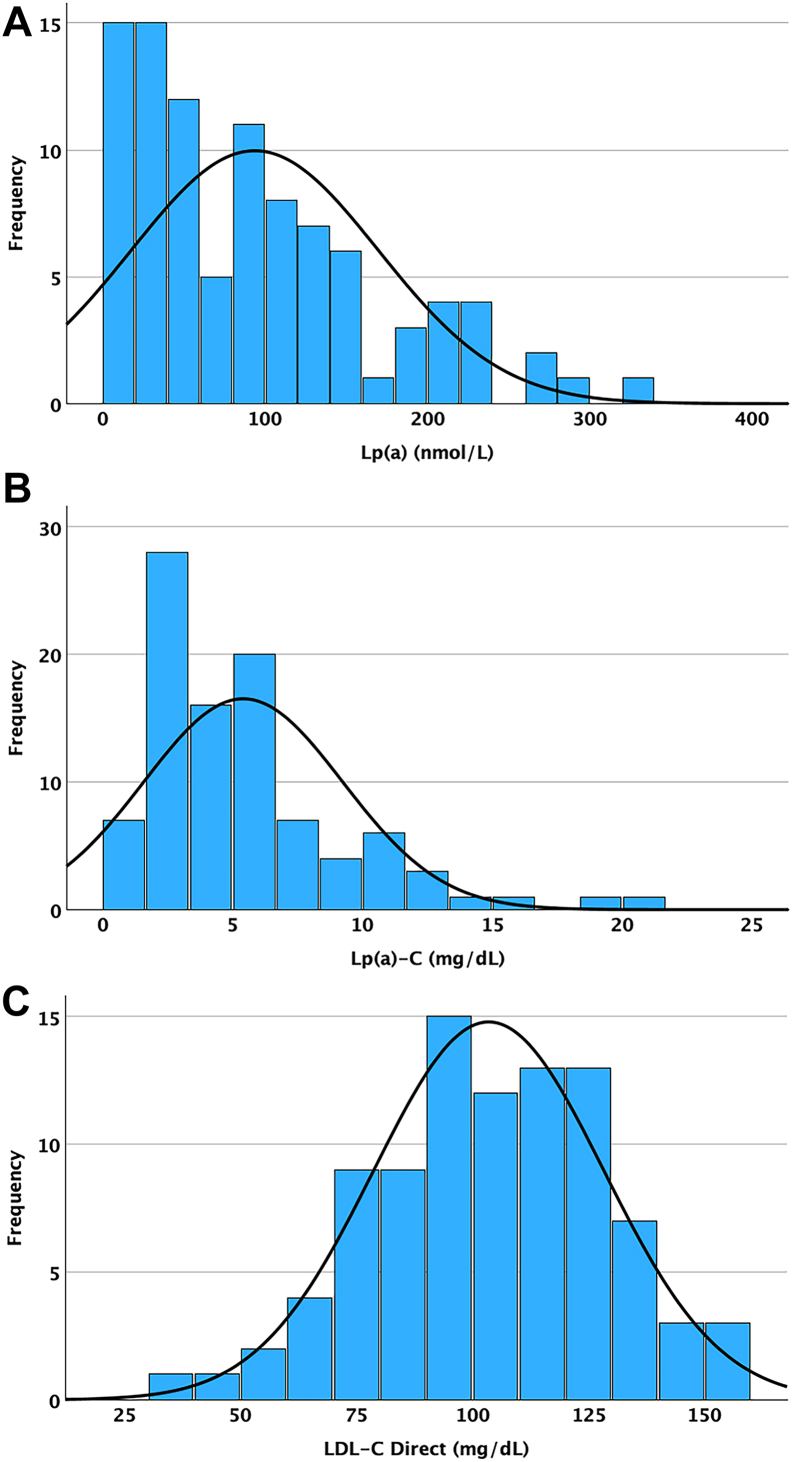
Frequency distribution of Lp(a) molar concentration (A) Lp(a)-C (B) and direct LDL-C (C) in healthy normolipidemic individuals.

The frequency distributions for Lp(a) molar concentration, direct Lp(a)-C, and direct LDL-C are shown in Figure 4. As expected, Lp(a) variables were right shifted, as were Lp(a)-C values, but LDL-C values were normally distributed.

### Correlations between Lp(a)-C, Lp(a) molar concentration, and LDL-C

**Figure 5:**
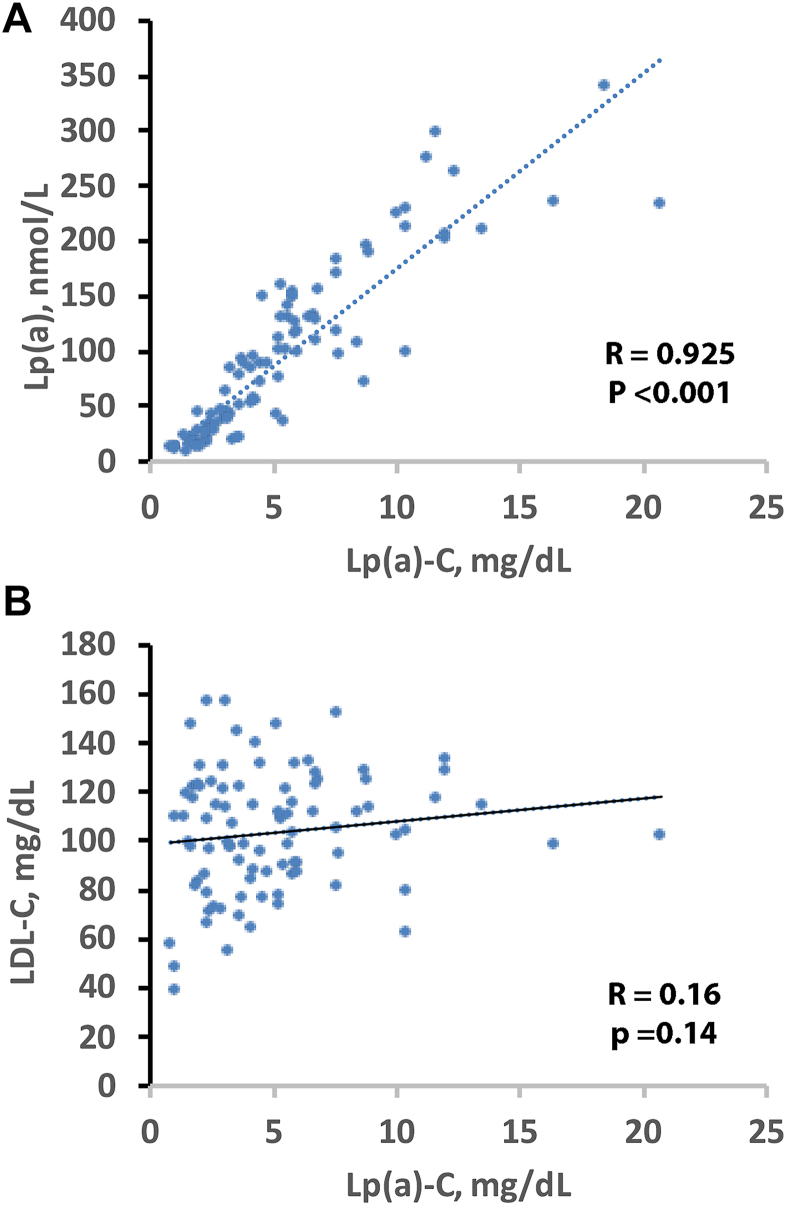
Relationships between Direct Lp(a)-C, Lp(a) and LDL-C. Scatterplots show the relationships between direct Lp(a)-C and Lp(a) molar (A) and LDL-C (B) in 94 healthy donors.

Lp(a) molar concentration demonstrated a very strong positive association with Lp(a)-C (ρ = 0.925, *P* < 0.001; Fig. 5A). In contrast, Lp(a)-C showed no meaningful association with directly measured LDL-C (ρ = 0.16, *P* = 0.14; Fig. 5B).

## Discussion

This study presents the analytical validation of the direct Lp(a)-C assay. The assay demonstrates high sensitivity, specificity, excellent linearity, and robust performance across a wide range of Lp(a) concentrations. Direct Lp(a)-C expected values in healthy individuals correlated strongly and increased linearly with Lp(a) molar concentration.

The direct Lp(a)-C method resolves inconsistencies introduced by the assumption of a fixed percent cholesterol content per Lp(a) particle, an assumption invalidated by the demonstrated variability in Lp(a)-C mass per particle (6). Prior attempts to estimate or quantify Lp(a)-C provided early evidence that Lp(a) contributes a significant amount of cholesterol, but the approaches showed several shortcomings. The Dahlén formula estimates Lp(a)-C by assuming that a fixed proportion of 30% of Lp(a) mass is cholesterol and subtracts this from LDL-C to derive a corrected value (10). However, this fixed proportion does not account for the individual variability in Lp(a)-C content, which can cause underestimation or overestimation of Lp(a)-C. A recent study proposed a calculated molar formula to estimate LDL-C corrected for Lp(a)-C; however, as the Dahlen formula, this approach does not account for the individual variability in Lp(a)-C and has not been biochemically validated against direct Lp(a)-C measurements across diverse populations (11). Densitometry-based methods for estimating Lp(a)-C are based on the separation of plasma lipoproteins by agarose gel electrophoresis. Enzymatic cholesterol staining produces visible bands, which are scanned by a densitometer, to generate a profile. The concentration of cholesterol in the presumed Lp(a) band is quantified as a percent area using image analysis multiplied by the total plasma cholesterol (12). However, these methods suffer from low sensitivity in quantifying Lp(a)-C values in subjects with low Lp(a), low specificity because of overlap between Lp(a) and LDL bands, poor reproducibility, high labor intensity, and low throughput.

The present study primarily included healthy donors with low-to-moderate Lp(a) concentrations, with mean Lp(a)-C concentrations of 5.4 mg/dl and a range of 0.8–20.7 mg/dl. In individuals with high Lp(a) (∼220–250 nmol/l), as observed in the various dose groups of the pelacarsen trials, in which Lp(a)-C was measured using the earlier research version of the immunocapture assay prior to the formal analytical validation reported here, the mean baseline Lp(a)-C values were 11.9–15.6 with a range of ∼3–39 mg/dl (6), a magnitude sufficient to potentially influence interpretation of LDL-C in clinical trial settings.

The direct Lp(a)-C assay may provide a platform for both research and clinical trial applications. First, it may offer mechanistic insight into lipoprotein remodeling by directly tracking how Lp(a)-C content changes with therapeutic interventions, diet, or metabolic states. Second, it may allow for endpoint refinement in lipid and cardiovascular outcome trials and serve as an independent pharmacodynamic marker to quantify the lipid effects of Lp(a)-lowering therapies. Expression of Lp(a)-C relative to apoB may further enhance mechanistic insight into particle-level remodeling and represents an important direction for future studies; however, apoB quantification was beyond the scope of the present analytical validation. While this study establishes analytical performance characteristics in a CLIA-certified laboratory, further validation in outcome-based studies will be necessary before routine clinical implementation in patient care.

This study has limitations. The plasma samples were obtained from a biobank of apparently healthy, fasting donors without available demographic or clinical data, precluding evaluation of age, sex, ethnicity, or metabolic influences on Lp(a)-C distribution. Because the primary objective of this study was analytical validation, the study did not include measurements of apoB or outcomes data linking Lp(a)-C or LDL-C_corr_ to cardiovascular risk. Future studies will extend these findings to larger and more diverse populations, integrating apoB and Lp(a)-C measurements to assess their associations with atherosclerotic burden, therapeutic response, and clinical outcomes.

In summary, this validated, high-throughput immunocapture assay provides a standardized approach to directly measure Lp(a)-C that may be used in future studies to define the role of Lp(a)-C and its impact on the interpretation of LDL-C, particularly in individuals with high Lp(a).

## Data availability

Data that support the plots within this publication and other findings of this study are available from the corresponding authors upon request.

### Supplemental data

This article contains Supplemental data.

## Conflict of interest

S. M. M. and S. S. are employees of Medpace Reference Laboratories. S. M. M. reports consulting roles for Denka. S. T. is a coinventor and receives royalties on patents held by the University of California on monoclonal antibodies directed to Lp(a) and oxidized phospholipids, is a cofounder and has an equity interest in Oxitope, Kleanthi Diagnostics, and Megaron, and has a dual appointment at the University of California San Diego and Ionis Pharmaceuticals. L. L. declares no conflicts of interest with the contents of this article.
