# Supplementary material for: Analytical validation of a direct lipoprotein(a)-cholesterol assay
Source: J Lipid Res. 2026 Feb 23;67(3):101008. doi: 10.1016/j.jlr.2026.101008 (PMC13022595; doi:10.1016/j.jlr.2026.101008)
Supplement: Supplemental Material [file mmc1.docx]

# Analytical Validation of Direct Lipoprotein(a)-Cholesterol Assay

# Santica M. Marcovina^1^, Spenser Smith^1^, Lizhu Lin^2^, Sotirios Tsimikas^2^ ^1^Medpace Reference Laboratories, Cincinnati, Ohio, USA ^2^Vascular Medicine Program, Division of Cardiology, University of California San Diego, La Jolla, California, USA

**Stability Assessment**

*Stability Assessment (Summary)*

- *Room Temperature (18–25 °C):*
  - *Stable up to 4 hours (%Bias < 20%).*
  - *Significant degradation observed at Day 1–7, not suitable for longer storage.*
  - *Recommendation: Process and freeze samples at −70 °C within 1 hour of collection.*
- *Refrigerated (4 °C):*
  - *Stable up to 4 hours (%Bias < 20%).*
  - *Instability observed beyond 4 hours (Day 1–7).*
  - *Recommendation: Same as above—freeze at −70 °C within 1 hour.*
- *Frozen Storage:*
  - *−20 °C: Stable for 3 months, though one sample at month 1 and another at month 3 exceeded 20% bias.*
  - *−70 °C: Stable for 3 month with an outlier sample at month 3.*
  - *Recommendation: Prefer long-term storage at −70 °C.*
- *Freeze–Thaw Cycles:*
  - *−20 °C: Instability after one freeze–thaw cycle.*
  - *−70 °C: Stable through two additional cycles after the initial thaw.*
- *Summary:
  Lp(a)-C in EDTA plasma is stable up to 4 hours at room temperature or 4 °C, and for at least 1 month when frozen at −70 °C. Extended or repeated freeze–thaw exposure should be avoided.*

*Full details below:*

*Baseline Results:* For each sample, six replicates were analyzed and the mean, SD, and %CV were calculated. Results are included in the table below. The %CV ranged from 7.0% to 15.8% for all six samples. All %CVs were within ±20.0% and therefore acceptable, so the mean value for each sample was used as the fresh day 0.

*Room Temperature (18 - 25°C) Results:* The mean %bias compared to fresh, day 0 ranged from -40.7% to 39.4% from day 1 to day 7. Therefore, the results did not meet the acceptance criteria.

*Room Temperature (18 - 25°C) Preanalytical Stability Results:* Since room temperature stability did not meet acceptance criteria, measurements were repeated with freshly prepared samples and the 4-hour time point was also included in addition to day 1, and day 7 time points. Compared to baseline, the mean %bias at hour 4 was -13.2% with a %Bias <20% for each individual sample with the exception of sample D which had a high %bias throughout all measurements. %Bias on individual samples at Day 1 and Day 7 did not meet the established criteria.

Room Temperature Conclusions: Lp(a)-C is stable for up to 4 hours in samples kept at room temperature. It is recommended the samples be processed and stored at -70°C within 1 hour from blood collection.

*Refrigerated (4°C) Results:* The mean %bias compared to fresh, day 0 did not meet the accepted criteria ranging from -22.4% to 51.1% from day 1 to day 7.

*Refrigerated (4°C) Preanalytical Stability Results:* Since refrigerated stability did not meet acceptance criteria, stability measurements were repeated to include hour 4, in addition to day 1, and day 7 time points. Compared to baseline, the mean %Bias ranged from -38.4% to 19.9%. While the %Bias at hour 4 was <20% on all individual samples, the %Bias exceeded 20% in several samples at Day 1 and in all the samples at Day 7.

Refrigerated (4°C) Conclusions: Lp(a)-C is stable in EDTA plasma samples when kept refrigerated for up to 4 hours. However, it is recommended that samples should be frozen at -70°C within 1 hour from blood collection.

*Frozen (-20°C) Results*: The mean %bias compared to fresh, day 0 results was 7.5% at month 1 and 9.5% at month 3 with only 1 sample slightly exceeding the 20% Bias criteria.

*Frozen (-70°C) Results:* The mean %bias compared to day 1 results was -0.7% at month 1 and 15.1% at month 3. One sample with an aberrant result at month 3 was considered an outlier with no samples exceeding the 20% bias criteria at either month.

Frozen (-20°C and -70°C) Conclusion: Lp(a)-C in EDTA plasma samples is stable for up to 3 months. Long-term stability testing is ongoing.

*Freeze/Thaw Stability at* -20°C and *-70°C:* For -20°C the mean %bias compared to the Day 1 result ranged from -7.2% to -16.5% but the %bias on several individual sample exceeded the 20% criteria at each F/T cycle. It is concluded that samples stored at -20°C cannot undergo additional freeze/thaw cycles after the initial thaw. For -70°C the mean %bias compared to the Day 1 result ranged from -10.9% to 15.2% through 3 F/T cycles. It is concluded that samples stored at -70°C can undergo two additional freeze/thaw cycles after the initial thaw.
